# Cardiac-specific CGI-58 deficiency activates the ER stress pathway to promote heart failure in mice

**DOI:** 10.1038/s41419-021-04282-7

**Published:** 2021-10-26

**Authors:** Xin Xie, Yi-Fan Tie, Song Lai, Yun-Long Zhang, Hui-Hua Li, Ying Liu

**Affiliations:** 1 Department of Cardiology, Institute of Cardiovascular Diseases, First Affiliated Hospital of Dalian Medical University, 116011 Dalian, China; 2 Department of Emergency Medicine, Beijing Key Laboratory of Cardiopulmonary Cerebral Resuscitation, Beijing Chaoyang Hospital, Capital Medical University, 100020 Beijing, China

**Keywords:** Heart failure, Heart failure

## Abstract

Excess myocardial triacylglycerol accumulation (i.e., cardiac steatosis) impairs heart function, suggesting that enzymes promoting triacylglycerol metabolism exert essential regulatory effects on heart function. Comparative gene identification 58 (CGI-58) is a key enzyme that promotes the hydrolysis of triglycerides by activating adipose triglyceride lipase and plays a protective role in maintaining heart function. In this study, the effects of CGI-58 on heart function and the underlying mechanism were investigated using cardiac-specific CGI58-knockout mice (CGI-58^cko^ mice). Echocardiography and pathological staining were performed to detect changes in the structure and function of the heart. Proteomic profiling, immunofluorescent staining, western blotting, and real-time PCR were used to evaluate molecular changes. In CGI-58^cko^ mice, we detected cardiac hypertrophic remodeling and heart failure associated with excessive cardiac lipid accumulation, ROS production, and decreased expression of regulators of fatty acid metabolism. These changes were markedly attenuated in CGI-58^cko^ mice injected with rAAV9-CGI58. A quantitative proteomics analysis revealed significant increases in the expression of ER stress-related proteins and decreases in proteins related to fatty acid and amino acid metabolism in the hearts of CGI-58^cko^ mice. Furthermore, the inhibition of ER stress by the inhibitor 4-PBA improved mitochondrial dysfunction, reduced oxidative stress, and reversed cardiac remodeling and dysfunction in cultured cardiomyocytes or in CGI-58^cko^ mice. Our results suggested that CGI-58 is essential for the maintenance of heart function by reducing lipid accumulation and ER stress in cardiomyocytes, providing a new therapeutic target for cardiac steatosis and dysfunction.

## Introduction

Cardiac energy metabolism is correlated with cardiac function and plays a critical role in the pathogenesis of heart failure (HF) [1]. In adult fasting mammals, the oxidation of fatty acids (FAs) contributes 60–80% of the energy used by the heart. The impairment of FA metabolism leads to cardiac dysfunction. FAs originate from myocardial triacylglycerol (TG) hydrolysis and provide ATP necessary for cardiac contraction [2], suggesting that TG hydrolysis exerts an essential role in regulation of cardiac function. Previous studies have revealed that myocardial TG accumulation is closely associated with cardiac myocyte apoptosis and contractile dysfunction in rodents and humans under different pathological conditions, such as ischemia and pressure overload [3–5]. Recent studies have verified that endoplasmic reticulum (ER) stress activation mediates lipid-driven cardiac dysfunction [6, 7]. In response to stress, ER chaperones, such as GRP78, are markedly upregulated, and induction of C/EBP homologous protein (CHOP) or activation of the c-Jun N-terminal kinase and/or caspase-12-dependent pathways promotes the initiation of apoptotic processes when ER stress is prolonged [8]. Importantly, ER stress frequently impairs mitochondrial function and reduces energy expenditure, leading to cardiomyocyte apoptosis in hypertrophic or failing hearts after pressure overload or ischemic injury [9–12]. However, the causal relationship between lipid accumulation-induced ER stress and cardiac dysfunction is not clearly established.

Comparative gene identification 58 (CGI-58, also known as ABHD5) is a lipid droplet (LD)-associated protein with a critical role in the regulation of TG hydrolysis by activating adipose triglyceride lipase (ATGL) [13]. Mutations in the *CGI-58* gene in humans are associated with neutral lipid storage diseases, characterized by the accumulation of TG in multiple tissues [14]. CGI-58 is significantly downregulated in mice with high-fat diet (HFD) [15]. CGI-58 knockout increases TG accumulation in coronary atherosclerotic lesions and the myocardium ultimately leading to cardiac dysfunction [16]. Moreover, CGI-58- or ATGL-deficient mice show an increase in lipid accumulation and reduction of mitochondrial oxidative activity accompanied with cardiomyopathy [17, 18]. A recent study has reported that CGI-58 levels are reduced in failing human hearts, and cardiac-specific CGI-58 overexpression protects against pressure-overload-induced HF via the proteolysis of HDAC4 [19]. Although the effects of CGI-58 on lipid metabolism are well established, its role in cardiomyocytes and ER stress activation are less clear.

In this study, we investigated the effect of CGI-58 in the heart and the potential mechanism using cardiac-specific CGI58-knockout mice (CGI-58^cko^ mice). Our results showed that CGI58 knockout induces cardiac dysfunction and hypertrophic remodeling likely by increasing lipid accumulation and ER stress. Thus, CGI-58 has an important role in maintaining cardiac function and may represent a new therapeutic target for cardiac steatosis and HF.

## Materials and methods

### Antibodies and reagents

Anti-Chop (2895), anti-phospho-elf2α (3398), anti-elf2α (5324), anti-Atf6 (65880), and anti-α-tubulin (3873) antibodies were purchased from Cell Signaling Technology (Danvers, MA). Anti-phospho-IRE1 (ab48187), anti-IRE1 (ab37073), anti-GRP78 (ab21685), anti-Galectin3 (Mac-2) (ab76245), and anti-*O*-GlcNAc (ab2739) antibodies as well as the ATP Assay Kit (ab83355) and Protein Carbonyl Kit (ab109414) were purchased from Abcam (Cambridge, MA). Tamoxifen was obtained from MedChemExpress (Monmouth Junction, NJ). 4-Phenylbutyric acid (4-PBA), wheat germ agglutinin (WGA), dihydroethidium (DHE), and Oil Red O were obtained from Sigma-Aldrich (St. Louis, MO). Palmitic acid (PA) and its solvent (vehicle) were purchased from Kunchuang Biotechnology (Xi’an, Shanxi, China), in which PA was coupled to FA-free bovine serum albumin (BSA) in a ratio of 2 mM PA:3% BSA. The TG Content Assay Kit, Total Cholesterol (TC) Content Assay Kit, and Hematoxylin–Eosin (H&E) and Masson’s Trichrome Staining Kits were purchased from Solarbio (Beijing, China). JC-1 was obtained from Beyotime Biotechnology (Hangzhou, China). MitoSOX, BODIPY 558/568 C_12_ (Red C12), BODIPY493/503, and TRIzol were obtained from Invitrogen (Carlsbad, CA). Adenovirus expressing small interfering RNA (siRNA)-CGI58, adeno-associated virus-9 expressing CGI-58 (rAAV9-CGI58), or green fluorescent protein (GFP) control were prepared by Hanbio Biotech (Shanghai, China). All primers used in our laboratory were purchased from Sangon Biotech (Shanghai, China). The proteomic analysis was performed by Jingjie PTM Biolab (Hangzhou, China).

### Generation of CGI-58^cko^ and CGI-58^fl/fl^ mice

Cardiac-specific CGI-58 knockout mice were generated by mating Myh6-Cre-ERT2Tg transgenic mice created in our laboratory with homozygous CGI-58-floxed mice. Then 8–12-week-old male Cre^+^ floxed (CGI-58^fl/fl^) mice were injected with tamoxifen (20 mg/kg) or its solvent for 5 days to obtain CGI58-cKO and CGI-58^fl/fl^ mice. Age- and sex-matched mice were assigned randomly to the experimental and control groups. The CGI-58^fl/fl^ mice and Cre^+^ non-floxed mice showed no significant differences in body weight, muscle TG levels, and survival rates compared with C57BL/6 wild-type mice (data not shown). Tamoxifen (5 mg/mL) was dissolved in regular corn oil solution containing 10% ethanol. All mouse procedures were approved by the Animal Care and Use Committee of the Dalian Medical University. The animal experiments conformed to the Guide for the Care and Use of Laboratory Animals published by the National Institutes of Health.

### Diets, rAAV9 injection, and 4-PBA administration

Mice at 8–10 weeks of age were fed a normal diet (ND) and HFD for 16 weeks. The survival rate was recorded. For the rAAV9-CGI58 group, CGI-58^fl/fl^ and CGI-58^cko^ mice were intravenously injected with rAAV9-CGI58 or rAAV9-Control (1 × 10^12^ genome-containing particles) via the tail vein for 3 weeks and were sacrificed after additional 3 months of HFD. For ER stress inhibitor administration, mice were fed a HFD for 8 weeks and intraperitoneally injected with 4-PBA sodium salt (80 mg/kg, dissolved in 0.9% saline) once every other day for 4 weeks. Controls were administered similar volumes of saline. Mice were sacrificed by an overdose of anesthetics. The hearts were rapidly removed and washed in cold 0.9% saline, quickly transferred into liquid nitrogen, and stored at −80 °C until use for real-time PCR and western blotting. Hearts were also fixed in formalin for histopathological analysis.

### Measurement of TG and TC contents in the heart

The TG and TC contents in the hearts and livers were estimated colorimetrically using commercial kits (Solarbio). At the end of the experiment, the heart was collected, homogenized, and lysed in ice-cold *n*-heptane/isopropyl alcohol (1:1) solution. Lipids were extracted by centrifugation at 10,000 × *g* for 10 min at 4 °C to obtain the total lipid contents. The hepatic TC and TG contents were measured according to the instructions provided with the kits. Values are expressed as µg/mg of tissue.

### Echocardiography

All mice were lightly anesthetized with 2% isoflurane and the heart rate was maintained at 450–550 per minute. Then the structure and cardiac contractile function of mice were evaluated by M-mode echocardiography using a 30 MHz probe (Vevo 770 System; VisualSonics, Toronto, ON, Canada). The cardiac function was evaluated by the left ventricular ejection fraction (EF%) and fractional shortening (FS%), which were calculated using the Vevo Analysis software, as described previously [20].

### Histopathology

The hearts were fixed in 4% buffered formalin, embedded in paraffin, and then cut into serial 5-µm sections for the histopathological analysis. H&E, Masson’s Trichrome, and WGA staining were performed according to the product instructions, as described previously [20]. The fibrotic area and cross-sectional area of myocytes in the heart were calculated using Image Pro Plus 3.0. For Oil Red O staining, tissue sections were stained in 0.3% Oil Red O stock solution at 65 °C for 8 min. Tissue sections were mounted with aqueous mounting medium before imaging. Images of each heart section were obtained at ×100 or ×200 magnification in 10–20 random fields. Images were analyzed using Image Pro Plus 3.0 (Nikon, Tokyo, Japan).

### Culture of neonatal rat cardiomyocytes (NRCMs) and treatments

Hearts in SD rats (born within 24 h) were quickly removed, collected in ice-cold phosphate-buffered saline (PBS), and washed with PBS three times to remove the blood. The hearts were cut into 10 pieces and incubated with 0.04% trypsin and 0.07% type II collagenase at 37 °C, as described previously [20]. The suspension medium was centrifuged at 1000 rpm for 5 min. After sedimentation, isolated cardiomyocytes and fibroblasts were collected and resuspended in new culture medium (DMEM/F12, 10% fetal bovine serum, and 1% penicillin and streptomycin) for 2 h of incubation at 37 °C and 5% CO_2_. A portion of NRCMs was seeded onto coverslips and infected with siRNA-control or siRNA-CGI58 at multiplicity of infection 50 for 24 h and then treated with saline or 4-PBA (5 mM) for 1 h following PA (400 μM) or its solvent treatment for another 24 h.

### Mitochondrial membrane potential (Δψm)

The Δψm in NRCMs was evaluated by JC-1 staining, as described previously [21]. Briefly, NRCMs were washed with PBS three times and then incubated with 2 µM JC-1 in PBS for 30 min in a cell incubator protected from light. Following aspiration, the cells were washed by warm DPBS three times and images were digitally obtained using a fluorescence microscope (Olympus, BX51, Tokyo, Japan).

### Measurement of FA, LDs, and reactive oxygen species (ROS) levels

To visualize FAs, freshly isolated NRCMs were grown on coverslips for 48 h, washed three times with PBS, and incubated with 1 μM BODIPY558/568 C12 (Red C12) in culture medium overnight. To label LDs, 200 ng/mL BODIPY 493/503 was added to the cells for imaging. Thus, FAs were tracked by red fluorescence and LDs were tracked by green fluorescence according to previously described methods [22]. For ROS detection, cells were treated with 300 nM MitoSOX for 20 min at 37 °C in the dark and washed with warm PBS three times. Red MitoSOX fluorescence representing mitochondrial ROS was observed by fluorescence microscopy (Olympus, BX51) according to a previous report [23].

### Measurement of intracellular ATP

The ATP levels in NRCMs after the indicated treatment were measured by luminometric methods according to the manufacturer’s instructions. In brief, cells were washed with PBS and lysed in 50 μL of lysis buffer on ice. After centrifugation at 600 rpm for 5 min at 4 °C, 50 μL of diluted substrate solution was added to the supernatant of each sample. Then the microplate was shaken for 5 min and covered in the dark for 10 min, the microplate luminometer was used to evaluate emitted light, and the ATP content was calculated according to the standard curve. The protein concentration in cell lysates was determined using a Bicinchoninic acid (BCA) Assay Kit. Finally, the ATP concentration was normalized to the protein levels.

### Real-time PCR analysis

Real-time PCR analyses of atrial natriuretic peptide (*ANP*), brain natriuretic peptide (*BNP*), β-myosin heavy chain (*β-MHC*), collagen I, collagen III, interleukin (*IL*)*-1β*, *IL-6*, *ATGL*, peroxisome proliferator-activated receptor α (*PPAR-α*), *LCAD*, *CPT1-m*, and *HMGSC-2* were performed as described previously [20]. Relative transcript levels in the experimental groups were compared by using the relative quantitative method; all mRNA levels were normalized against the internal control (*GAPDH*). Values relative to those in the control sample were obtained by 2^−ΔΔ*CT*^. The primer sequences for each gene are shown in Table 1.

**Table 1:** Sequences of RT-PCR primers.

Gene name	Species	Forward primer(5’→3’)	Reverse primer (5’→3’)
ANP	Mouse	TACAGTGCGGTGTCCAACACAG	TGCTTCCTCAGTCTGCTCACTC
BNP	Mouse	TCCTAGCCAGTCTCCAGAGCAA	GGTCCTTCAAGAGCTGTCTCTG
β-MHC	Mouse	ACTGTCAACACTAAGAGGGTCA	TTGGATGATTTGATCTTCCAGGG
Collagen I	Mouse	GAGTACTGGATCGACCCTAACCA	GACGGCTGAGTAGGGAACACA
Collagen III	Mouse	TCCCCTGGAATCTGTGAATC	TGAGTCGAATTGGGGAGAAT
IL-1β	Mouse	GCAACTGTTCCTGAACTCAACT	ATCTTTTGGGGTCCGTCAACT
IL-6	Mouse	TACCACTTCACAAGTCGGAGGC	CTGCAAGTGCATCATCGTTGTTC
ATGL	Mouse	GACAGCTCCACCAACATCCA	GCAAAGGGTTGGGTTGGTTC
PPAR-α	Mouse	CATTGGTGTTCGCAGCTGTT	CCGGTGAGATACGCCCAAAT
LCAD	Mouse	AAACGTCTGGACTCCGGTTC	GTACCACCGTAGATCGGCTG
CPT1-m	Mouse	TGTCTACCTCCGAAGCAGGA	CCATGACCGGCTTGATCTCT
HMGSC-2	Mouse	AGTCCAGCTACTGGGATGGT	ACGCGTTCTCCATGTGAGTT

### Western blot analysis

Cardiac tissues were lysed in RIPA buffer containing 1% protease inhibitor cocktail for 15 min on ice. Samples were broken using ultrasound for 5 min and centrifuged at 10,000 × *g* for 15 min at 4 °C. After the supernatant in each sample was collected, the protein concentrations were quantified using the BCA method (Bio-Rad, Hercules, CA). Then the protein samples were separated by sodium dodecyl sulfate-polyacrylamide gel electrophoresis (SDS-PAGE) and transferred to polyvinylidene difluoride (PVDF) membranes. The membrane was blocked in 5% defatted milk for 1 h at room temperature and incubated with the appropriate antibodies at 4 °C overnight. All antibodies against Grp78ip, p-IRE, IRE, p-elf2a, elf2a, Atf6, Chop, O-GlcNAc, protein carbonyls, Acaa2, Hadh, and Tubulin were used as recommended by the manufacturer. After incubation with the secondary antibody and washing, the targeted protein was detected by using an ECL Advanced Western Blotting Kit and band densities were quantified using ImageJ. Phosphorylated protein levels were normalized against the total protein and then against tubulin levels. Other protein levels were normalized against tubulin. For multiple group comparisons, protein expression levels in other groups were normalized against the levels in CGI-58^fl/fl^ mice.

### Protein carbonylation assay

Protein carbonylation by oxidative stress in cardiac tissue was detected using a commercially available kit (Abcam, ab178020). Frozen tissue was powdered under liquid nitrogen and then cytosolic proteins were derivatized with dinitrophenylhydrazine (DNPH) under acid denaturing conditions. Next, protein samples were separated by 10% SDS-PAGE and transferred to PVDF membranes. Membranes were incubated with the primary antibody for DNPH overnight at 4 °C, followed by incubation with the secondary horseradish peroxidase-conjugated antibody for 1 h at room temperature and chemiluminescent detection using an ECL Advanced Western Blotting Kit. The band densities were quantified using Image J.

### Statistical analysis

No statistical methods were used to predetermine sample size. All data are presented as mean ± SEM. Statistical analyses were performed using GraphPad Prism 7. For pairwise comparisons, an unpaired two-tailed Student’s *t* test was performed; for multiple comparisons, one-way analysis of variance with post hoc Bonferroni corrections was used. The Kaplan–Meier method (log-rank test) was used for the survival analysis. A *P* value of <0.05 was considered statistically significant. For the proteomic analyses of hearts in CGI-58^cko^ and CGI-58^f/f^ mice, the “clusterProfiler” package in R was used for a Gene Ontology (GO) enrichment analysis [24] and DAVID (v6.8) was used for a Kyoto Encyclopedia of Genes and Genomes (KEGG) pathway enrichment analysis [25]. *P* values for both GO and KEGG categories were adjusted using the Benjamini–Hochberg method (false discovery rate *q* ≤ 0.05).

## Results

### CGI-58 deficiency in cardiomyocytes induces cardiac remodeling and dysfunction in mice fed a ND and HFD

**Fig. 1:**
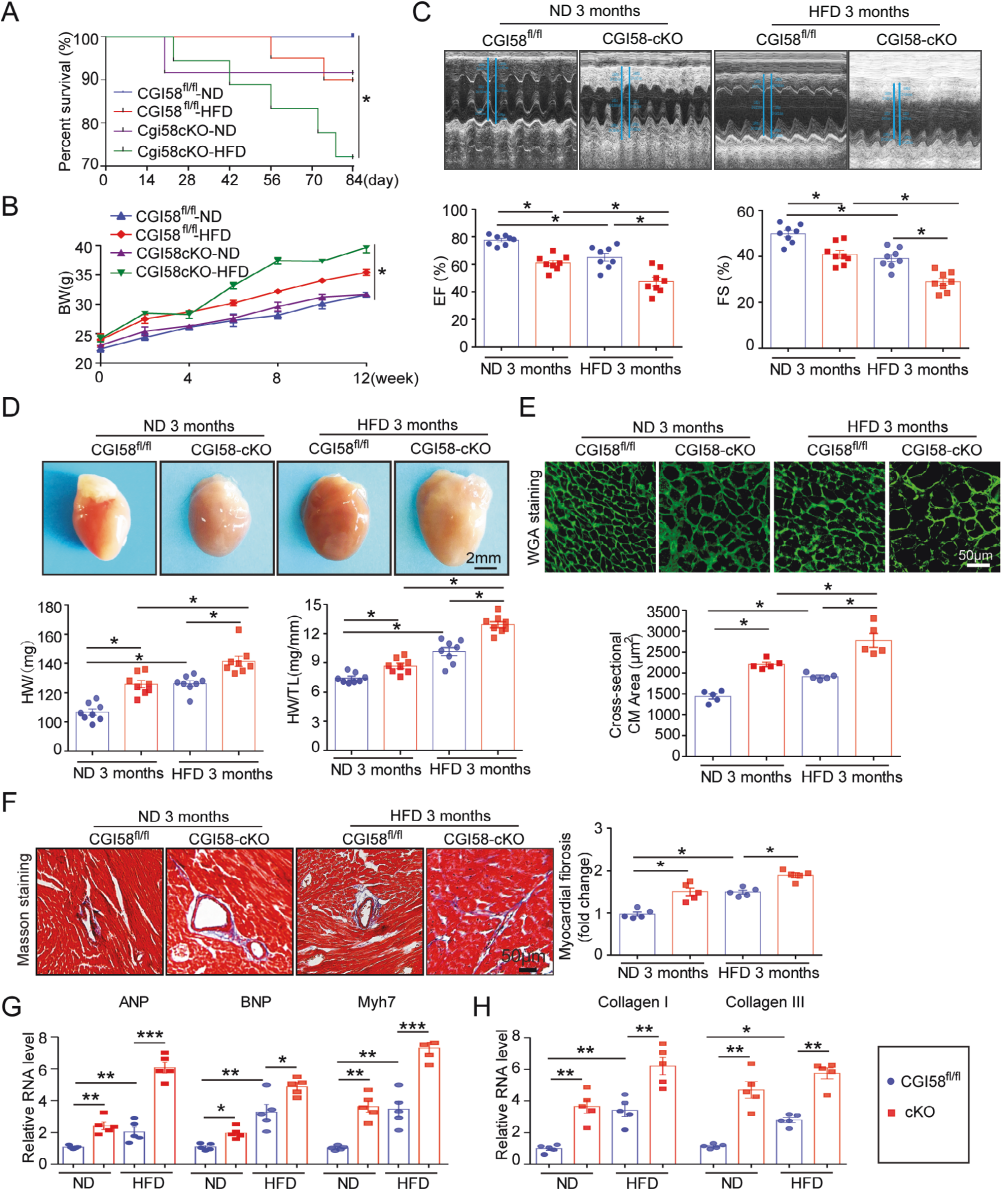
CGI-58-deficient mice develop cardiac hypertrophic remodeling and dysfunction. CGI-58^fl/fl^ and CGI-58^cko^ mice were fed with ND or HFD for 3 months. **A**, **B** The survival rate and body weight of CGI-58^fl/fl^ and CGI-58^cko^ mice (*n* = 10). **C** M-model echocardiography of the left ventricle (upper). Measurement of ejection fraction (EF%) and fractional shortening (FS%) (lower, *n* = 8). **D** Heart size (upper), heart weight, and heart weight to tibia length (HW/TL) ratio (lower, *n* = 8). Scale bar 2 mm. **E** Wheat germ agglutinin (WGA) staining of heart sections (upper) and the quantification of cardiac myocyte size (lower). Scale bar 50 μm (*n* = 5). **F** Masson’s Trichrome staining of heart sections (upper) and the quantification of the relative fibrosis area (lower, *n* = 5). **G**, **H** qPCR analyses of *ANF*, *BNP, Myh7*, *collagen I* and *collagen III* mRNA levels (*n* = 5). Data are presented as mean ± SEM, and *n* represents the number of animals per group. *P < 0.05, **P < 0.01, ***P < 0.001.

To investigate whether CGI-58 regulates heart function, we selectively reduced CGI-58 expression in cardiomyocytes of mice using the Cre-LoxP system and confirmed the downregulation of CGI-58 expression in the heart by immunoblotting (Supplemental Fig. 1). ATGL protein expression was also reduced in CGI-58^cko^ mice (Supplemental Fig. 1). Two-month-old cardiomyocyte-specific CGI-58 knockout (CGI-58^cko^) mice and homozygous floxed (CGI-58^fl/fl^) littermates were fed a ND and HFD for 3 months. Compared with CGI-58^fl/fl^ controls, the survival rates of CGI-58^cko^ mice were reduced by about 9 and 28%, respectively, after ND and HFD (Fig. 1A). Weight gain was similar in the two groups during 3 months of chow diet feeding, but the HFD induced significantly greater weight gain in CGI-58^cko^ mice than in CGI-58^f/f^ mice (Fig. 1B). Furthermore, CGI-58^cko^ mice showed a significant decrease in left ventricular (LV) contractile function, as indicated by a decreased LV EF% and FS% compared with those in CGI-58^f/f^ mice under ND conditions (Fig. 1C). Interestingly, the HFD further aggravated cardiac dysfunction in both CGI-58^f/f^ and CGI-58^cko^ mice (Fig. 1C). CGI-58^cko^ mice developed marked cardiac hypertrophy, as evidenced by an increased heart size, heart weight to tibial length (HW/TL) ratio, and cross-sectional area of myocytes compared with those in CGI-58^fl/fl^ controls under both ND and HFD conditions, and these changes were more obvious in HFD-fed mice than in ND-fed mice (Fig. 1D, E). In addition, as determined by Masson’s Trichrome staining, CGI-58^cko^ mice also showed a marked increase in cardiac fibrosis compared with CGI-58^fl/fl^ controls under both ND and HFD conditions (Fig. 1F). Consistent with these data, the mRNA levels of hypertrophic markers (*ANP, BNP*, and *Myh7*) and fibrotic markers (collagen I and collagen III) were higher in CGI-58^cko^ mice than in CGI-58^fl/fl^ controls under both ND and HFD conditions (Fig. 1G, H).

### CGI-58 knockout increases lipid accumulation, ROS production, and inflammation in the heart

**Fig. 2:**
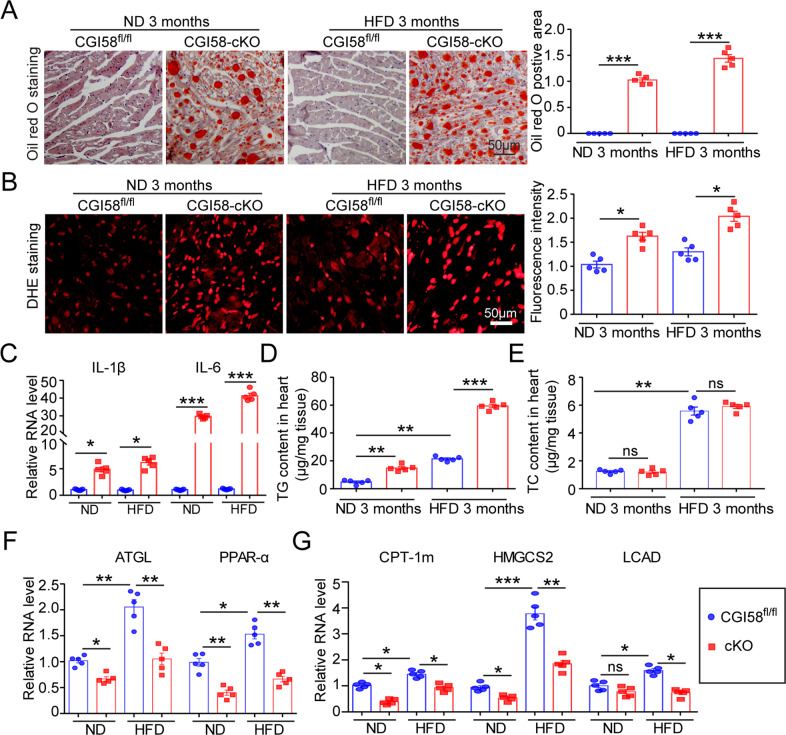
CGI-58 deficiency induces lipid accumulation, oxidative stress, and inflammation in the hearts of ND and HFD-fed mice. CGI-58^fl/fl^ and CGI-58^cko^ mice were fed with ND and HFD for 3 months. **A** Oil Red O staining of the heart to measure lipid accumulation (*n* = 5). **B** DHE staining of the heart sections (left) and the quantification of ROS level (right, *n* = 5). Scale bar 50 μm. **C** qPCR analyses of IL-1β and IL-6 mRNA expression (*n* = 5). **D**, **E** Measurement of TG and TC content in the heart (*n* = 5). **F**, **G** qPCR analyses of ATGL, PPARα, CPT-1m, HMGCS2, and LCAD mRNA levels (*n* = 5). Data are presented as mean ± SEM, and *n* represents the number of animals per group. *P < 0.05, **P < 0.01, ***P < 0.001.

To test whether CGI-58 deficiency-mediated cardiac remodeling and dysfunction were accompanied by increased lipid accumulation, oxidative stress, and inflammatory responses, we performed Oil Red O staining, DHE staining, and real-time PCR. The Oil Red-positive area, ROS level, and expression levels of the proinflammatory cytokines IL-1β and IL-6 in CGI-58^cko^ mice were significantly higher than those in CGI-58^f/f^ mice after 3 months of both ND and HFD feeding (Fig. 2A–C). In agreement with the increased lipid accumulation, the TG and TC contents were significantly higher in the hearts of CGI-58^cko^ mice than in CGI-58^f/f^ mice (Fig. 2D, E). However, there were no significant differences in total lipid, TG, and TC contents between the livers of CGI-58^f/f^ and CGI-58^cko^ mice, indicating that CGI-58 knockout in the heart did not affect lipid metabolism in the liver (Supplemental Fig. 2). The mRNA levels of *ATGL*, *PPAR-α*, *CPT1-m*, *HMGSC2*, and *LCAD*, which are involved in the regulation of FA metabolism, were all markedly upregulated in HFD-fed mice compared with ND-fed mice and were slightly or markedly downregulated in CGI-58^cko^ mice compared with CGI-58^f/f^ mice under both ND and HFD conditions (Fig. 2F, G).

### Restoration of CGI-58 in cardiomyocytes improves cardiac function

**Fig. 3:**
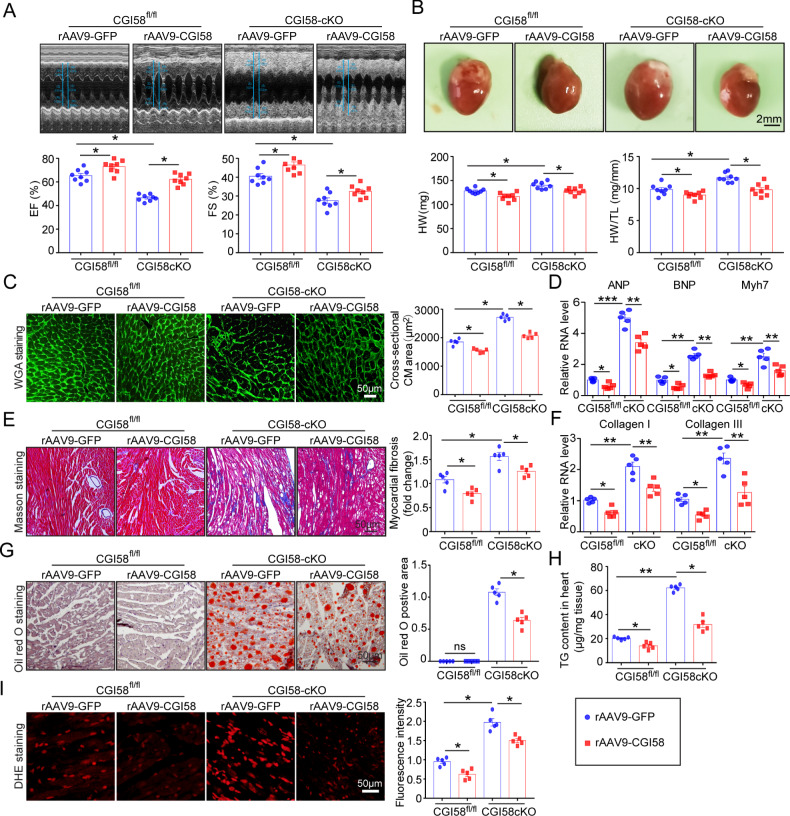
Re-expression of CGI-58 by rAAV9 injection reverses cardiac remodeling and dysfunction in CGI-58 knockout mice. **A** M-model echocardiography of the left ventricle (upper). Measurement of EF% and FS% (lower, *n* = 8). **B** Heart size (upper), heart weight, and HW/TL ratio (lower, *n* = 8). Scale bar 2 mm. **C** FITC-labeled WGA staining of the heart sections (upper). Quantification of the relative myocyte cross-sectional area (lower, *n* = 5). **D** qPCR analyses of *ANP*, *BNP*, and *Myh7* mRNA levels (*n* = 5). **E** Masson’s Trichrome staining of heart sections (left) and the quantification of fibrotic area (right, *n* = 5). **F** qPCR analyses of *collagen I* and *collagen III* mRNA levels (*n* = 5). **G** Oil Red O staining of heart sections (left) and the quantification of lipid accumulation (*n* = 5). **H** Measurement of TG content in the heart (*n* = 5). **I** DHE staining of heart sections (left) and the quantification of ROS level (right, *n* = 5). Scale bar 50 μm. Data are presented as mean ± SEM, and *n* represents the number of samples or animals. *P < 0.05, **P < 0.01, ***P < 0.001.

To assess whether restoring CGI-58 expression in cardiomyocytes alleviates cardiac remodeling and dysfunction, CGI-58^f/f^ and CGI-58^cko^ mice were randomized to receive rAAV9-CGI-58 (1 × 10^12^) or an equivalent amount of empty rAAV9-GFP particles for 3 weeks and then fed an HFD for an additional 3 months. Immunoblotting revealed that the injection of rAAV9-CGI58 into CGI-58^cko^ mice for 3 weeks resulted in markedly higher CGI58 protein expression levels in the heart than those in rAAV9-GFP controls (Supplemental Fig. 3). Moreover, rAAV9-CGI58 injection significantly improved cardiac dysfunction (EF% and FS%) assessed by echocardiography, attenuated cardiac hypertrophy, and fibrosis (reduced heart size, HW and HW/TL ratio, cross-sectional area of myocytes, and mRNA levels of *ANP*, *BNP*, *Myh7*, collagen I, and collagen III) compared with rAAV9-empty injection in both CGI-58^f/f^ and CGI-58^cko^ mice (Fig. 3A–F). Furthermore, the accumulation of lipids, TG content, and ROS levels were much lower in rAAV9-CGI58-injected mice than in rAAV9-empty-injected mice (Fig. 3G–I).

### Proteomic profiling of the heart in CGI-58^cko^ mice

**Fig. 4:**
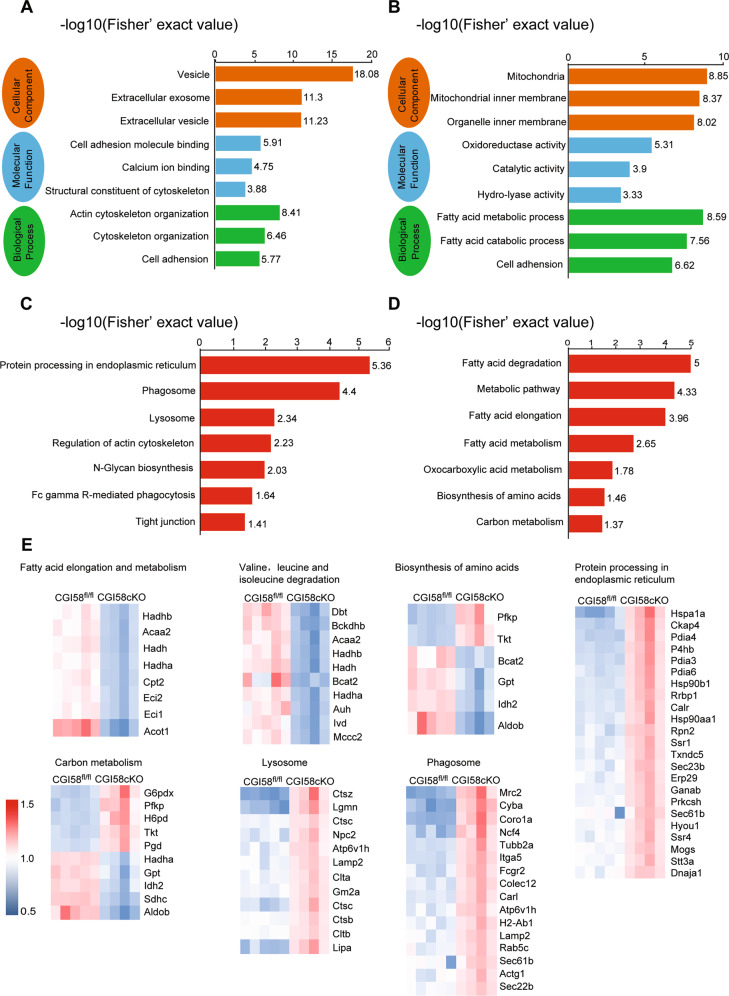
Proteomic exploration of the biological processes of CGI-58^cko^ heart. **A**–**D** GO and KEGG pathway analysis of the biological processes in the hearts from CGI-58^fl/fl^ and CGI-58^cko^ mice. The pathways involved in ER stress, phagosome, lysosome, and fatty acid metabolism are significantly enriched in CGI-58^cko^ hearts. The horizontal axis represents −log10 (Fisher’ exact value); the ordinate represents the GO or KEGG pathway. **E** The heatmap illustrates the expression level changes of the statistically significant proteins involved in ER stress, phagosome, lysosome, and fatty acid metabolism between the CGI-58^fl/fl^ and CGI-58^cko^ mice.

To elucidate the molecular mechanisms by which CGI-58 knockout aggravates cardiac hypertrophy, we conducted proteomic analyses of CGI-58^cko^ mice and littermates fed the HFD. Using the DAVID functional annotation tool, enriched GO annotations in the three major categories, cellular components, molecular functions, and biological processes, were identified (Fig. 4A, B). Furthermore, KEGG pathway enrichment analysis revealed that the two most highly enriched pathways were “protein processing in endoplasmic reticulum (ER)” and “FA degradation” (Fig. 4C, D), which are closely related to cardiac dysfunction and pathological remodeling. We further analyzed the enriched biological processes and signaling pathways for differentially expressed genes between the hearts of CGI-58^fl/fl^ and CGI-58^cko^ mice. Various proteins related to lysosomes, phagosomes, and protein processing in the ER were upregulated in CGI-58^cko^ hearts, whereas proteins that regulate FA metabolism were downregulated, suggesting that CGI-58^cko^ hearts exhibit ER stress and FA accumulation (Fig. 4E).

### CGI-58 knockout induces ER stress, oxidative stress, protein glycosylation, and lipid accumulation

**Fig. 5:**
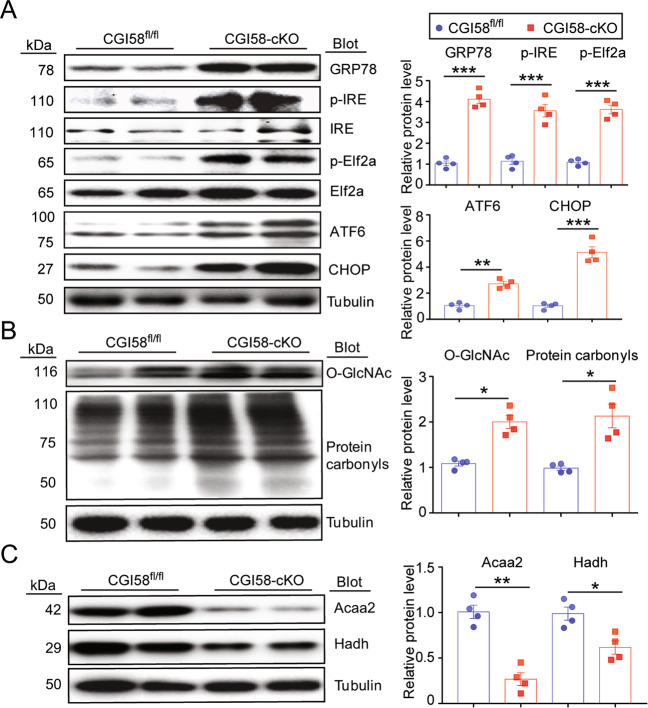
CGI-58 knockout influences ER stress, oxidative stress, protein glycosylation, and fatty acid oxidation in the heart. **A**–**C** CGI-58^fl/fl^ and CGI-58^cko^ mice were fed with ND for 3 months, and then the protein levels of GRP78, p-IRE, p-Elf2a, ATF6, CHOP, O-GlcNAc, protein carbonyls, Acaa2, and Hadh in the heart were examined. All statistical results are shown right, *n* = 4 per group. Data are presented as mean ± SEM, and *n* represents the number of samples or animals. *P < 0.05, **P < 0.01, ***P < 0.001.

To confirm the results of the proteomic analysis in CGI-58^cko^ mice, we examined the expression levels of proteins that regulate ER stress, oxidative stress, protein glycosylation, and lipid metabolism. We found that CGI-58 knockout significantly upregulated the protein levels of GRP78, p-IRE, p-Elf2a, ATF6, and CHOP, indicating that CGI-58 knockout activated ER stress in the heart (Fig. 5A). *O*-GlcNAc and carbonyls, indicators of oxidative stress and protein glycosylation, were also markedly increased in the hearts of CGI-58^cko^ mice compared with CGI-58^fl/fl^ controls (Fig. 5B). Of note, levels of both Acaa2 and Hadh, which promote FA oxidation, were reduced in CGI-58^cko^ mouse hearts (Fig. 5C). These results confirm that CGI-58^cko^ mice exhibit increased ER stress and oxidative stress and the inhibition of FA oxidation, and these changes in protein expression were consistent with the proteomic profiling results (Fig. 4).

### Inhibition of ER stress by 4-PBA improves mitochondrial dysfunction and oxidative stress in CGI-58-knockdown NRCMs

**Fig. 6:**
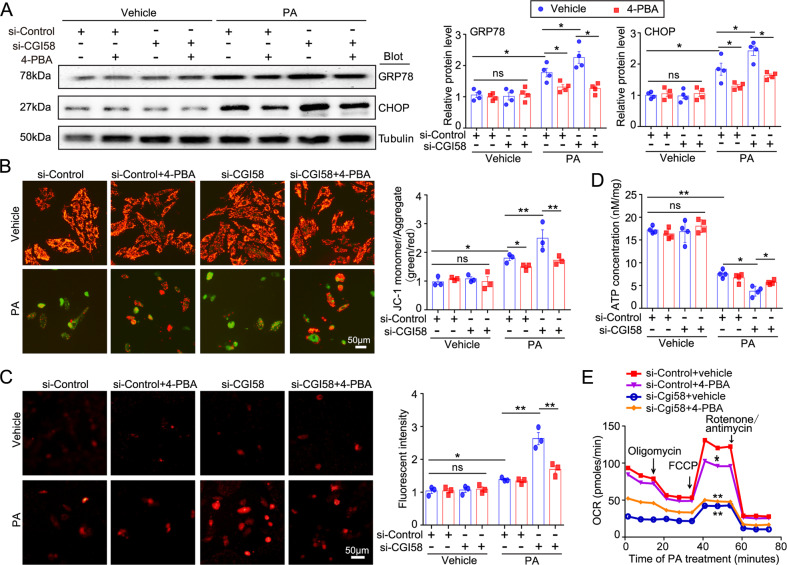
ER stress inhibitor 4-PBA attenuates ROS generation and mitochondrial dysfunction in CGI-58 knockdown NRCMs. **A** Western blot analysis of GRP78 and CHOP protein levels (*n* = 4). **B** JC-1 staining of mitochondrial membrane potential (left), and the ratio of JC-1 in the polarized or aggregate form (red) to the depolarized or monomer form (green) (right, *n* = 3). **C** The ROS level in NRCMs was evaluated with MitoSOX (0.5 mM; left), and the quantification of MitoSOX fluorescence from 100 cells of each independent experiment (right, *n* = 3). **D** The ATP content in NRCMs (*n* = 4). **E** Cardiomyocyte OCR changes examined by Seahorse XF 24 extracellular analyzer. *P < 0.05, **P < 0.01.

Since ER stress was activated in the hearts of CGI-58^cko^ mice (Figs. 4 and 5A), we next investigated whether blocking ER stress alleviates mitochondrial dysfunction and restores ATP production in vitro. NRCMs were transfected with si-Control or si-CGI-58 for 24 h and then treated with or without 4-PBA (an inhibitor of ER stress) in the presence of PA (a common saturated free fatty) or its solvent control for another 24 h. PA treatment upregulated the protein levels of GRP78 and CHOP in si-Control-transfected NRCMs compared with vehicle control, and si-CGI-58 transfection aggravated this effect (Fig. 6A). However, 4-PBA treatment inhibited the PA-induced upregulation of GRP78 and CHOP in si-Control- or si-CGI-58-transfected NRCMs (Fig. 6A), indicating that 4-PBA inhibits ER stress. Interestingly, 4-PBA treatment had no effect on CGI-58-knockdown-triggered LD accumulation (Supplemental Fig. 4) but significantly inhibited PA-induced mitochondrial depolarization (JC-1 fluorescence staining) in si-Control- or si-CGI-58-transfected NRCMs (Fig. 6B). The PA-induced increase in ROS generation (determined by MitoSOX staining) and decrease in intracellular ATP level were markedly reversed by 4-PBA in si-CGI-58-transfected NRCMs but not in si-Control-transfected cells (Fig. 6C, D). Unexpectedly, 4-PBA treatment significantly reduced oxygen consumption rat (OCR) in si-Control-transfected NRCMs and had no effect on OCR in si-CGI-58-transfected NRCMs after PA treatment (Fig. 6E). There were no differences in these parameters (GRP78, CHOP, mitochondrial depolarization, ROS, and ATP levels) between the two groups after vehicle treatment in the presence or absence of 4-PBA (Fig. 6A–D).

### Blocking ER stress by 4-PBA attenuates cardiac dysfunction, hypertrophy, fibrosis, lipid accumulation, and ROS production in CGI58-knockout mice

**Fig. 7:**
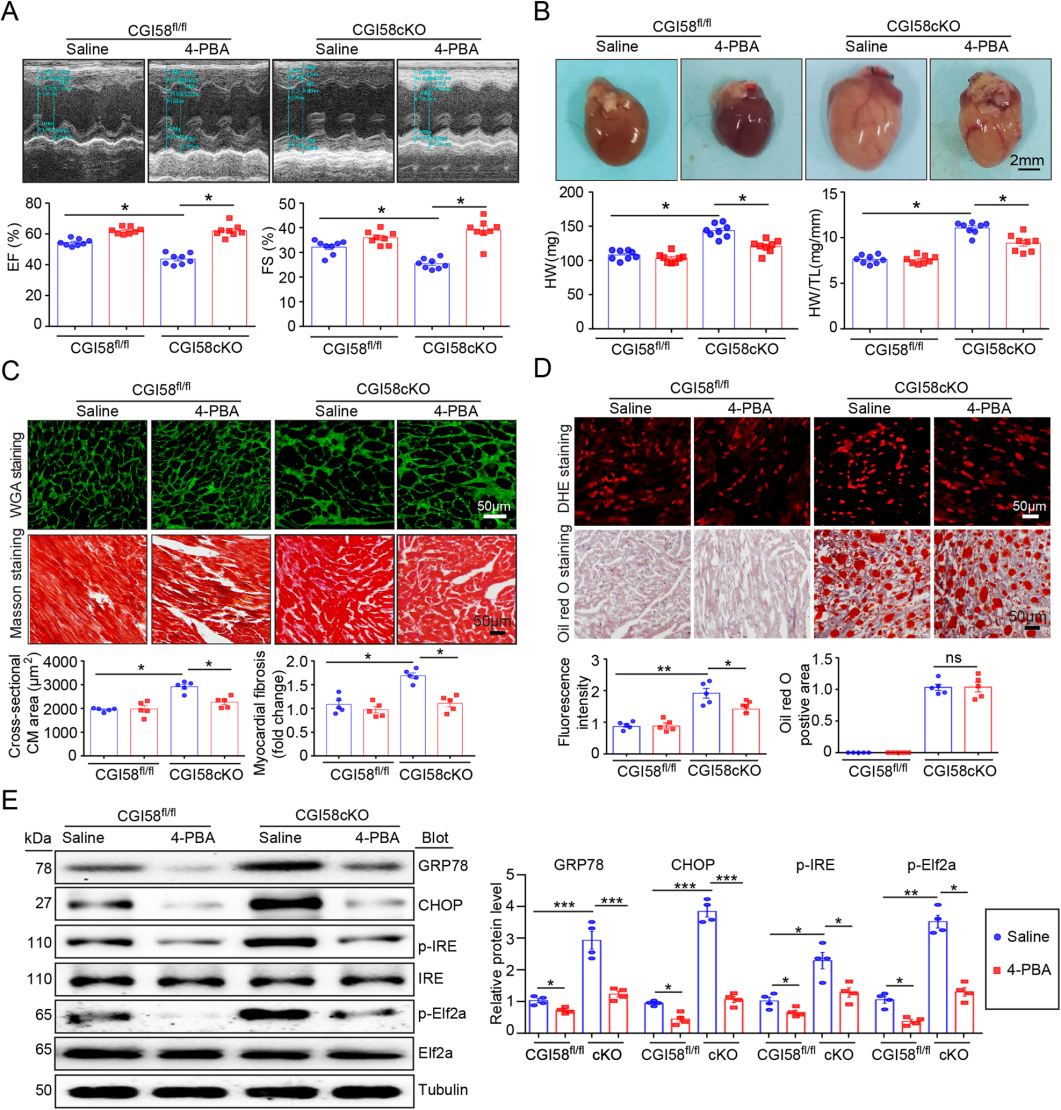
Administration of 4-PBA inhibits cardiac hypertrophic remodeling and dysfunction in CGI-58^cko^ mice. **A** M-model echocardiography of the left ventricle (upper). Measurement of EF% and FS% (lower, *n* = 8). **B** Heart size (upper), heart weight, and HW/TL ratio (lower, *n* = 8). Scale bar 2 mm. **C** FITC-labeled WGA staining or Masson’s Trichrome staining of the heart sections (upper). Quantification of the relative myocyte cross-sectional area and fibrotic area (lower, *n* = 5). **D** DHE or Oil Red O staining of the heart sections (upper), and the quantification of ROS and lipid levels (lower, *n* = 5). Scale bar 50 μm. **E** Western blot analysis of GRP78, CHOP, p-IRE, and p-Elf2a protein levels (left), and the quantification of relative protein level (right, *n* = 4). Data are presented as mean ± SEM, and *n* represents the number of samples or animals. *P < 0.5, **P < 0.01, ***P < 0.001.

We then hypothesized that inhibiting ER stress by 4-PBA also prevents CGI-58-knockout-induced cardiac hypertrophy in mice. To test this hypothesis, CGI-58^cko^ mice were treated with the ER stress inhibitor 4-PBA (80 mg/kg, once every other day) for 4 weeks. We found that the administration of 4-PBA significantly reversed contractile dysfunction, as indicated by the increased EF% and FS% (Fig. 7A), and markedly inhibited cardiac hypertrophy (reduced heart size, HW and TL/BW ratio, and myocyte cross-sectional area) and fibrosis in CGI-58^cko^ mice compared with saline-treated CGI-58^cko^ mice (Fig. 7B, C). Moreover, 4-PBA treatment remarkedly reduced ROS generation. However, it had no effect on lipid accumulation in the heart of CGI-58^cko^ mice compared with that in saline-treated CGI-58^cko^ mice (Fig. 7D). Moreover, 4-PBA inhibited ER stress, as evidenced by decreased protein levels of GRP78, CHOP, p-IRE, and p-Elf2a in CGI-58^cko^ mice than in saline-treated CGI-58^cko^ mice (Fig. 7E). These results demonstrated that CGI-58 knockdown promoted cardiac dysfunction and hypertrophic remodeling mainly by linking lipid accumulation and ER stress.

## Discussion

**Fig. 8:**
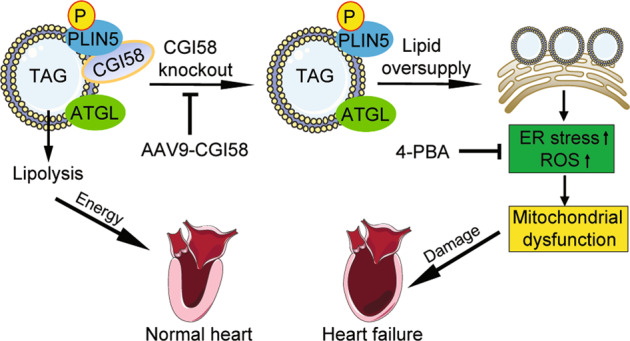
A working model for CGI-58 to regulate cardiac function. Under normal condition, CGI-58 binds and activates ATGL to promote lipolysis, which then provides energy to the heart. Conversely, CGI-58 knockout inhibits lipolysis and increases lipid accumulation in ER, which results in activation of ER stress and excessive ROS production thereby leading to mitochondrial dysfunction and heart failure. Blocking ER stress reverses mitochondrial dysfunction, ROS production, and cardiac dysfunction.

Here, using cardiac-specific knockout mice, we demonstrated that CGI-58 deficiency has a crucial role in regulating cardiac hypertrophy and dysfunction mainly by increasing lipid accumulation, ER stress, ROS generation, and impairing mitochondrial function. Importantly, inhibition of ER stress ameliorated HFD-induced cardiac dysfunction and hypertrophic remodeling accompanied by improved mitochondrial dysfunction. Overall, these results suggest that *CGI-58* is necessary for maintaining cardiac function and is a potential therapeutic target for cardiac steatosis and HF. These data are summarized in Fig. 8.

CGI-58 and ATGL promote lipid hydrolysis, which plays critical roles in the regulation of TG catabolism, mitochondrial oxidation of FAs, and ATP and ROS generation in cardiomyocytes. Deficiency of ATGL in the heart causes excess lipid accumulation and severely impaired contractile function in mice [17, 26]. Conversely, ATGL overexpression improved cardiac lipid metabolism and cardiac dysfunction [27]. Similarly, CGI-58 deficiency in the muscle also markedly increases TG accumulation and reduces mitochondrial FA oxidative activity, leading to severe cardiac steatosis and cardiomyopathy [16]. Interestingly, CGI-58 levels are downregulated in failing human hearts. Cardiac-specific overexpression of CGI-58 in mice reduces intra-cardiomyocyte lipid accumulation and prevents pressure-overload-induced HF [19]. Our present study extended these findings, revealing that a cardiac-specific CGI-58 deficiency significantly aggravated cardiac hypertrophic remodeling and contractile dysfunction accompanied by increased TG levels, ROS production, protein glycosylation, and oxidation, whereas the expression levels of regulators of FA metabolism (ATGL, PPARα, CPT-1m, HMGCS2, and LCAD) were reduced in the heart of CGI-58^cko^ mice under ND and HFD conditions (Figs. 2 and 5). However, these effects were restored in CGI-58^cko^ mice injected with rAAV9-CGI-58 (Fig. 3). It should be emphasized that our data were obtained from cardiac-specific CGI-58-deficient mice (CGI-58^cko^ mice) and better explain the effect of CGI-58 on the heart.

It has been reported that severe cardiac lipotoxicity contributes to HF [28, 29]. Of note, ER stress is the most important mechanism underlying lipotoxicity-induced HF [30, 31]. The activation of ER stress signaling subsequently leads to mitochondrial dysfunction and reduces energy expenditure, inflammatory response, and cell apoptosis [32, 33]. Although similar pathological phenotypes to those of CGI-58-knockout mice have been reported in recent studies [18, 19], the mechanisms underlying these phenotypic changes were quite different. By proteomic analyses, we identified a range of proteins involved in ER stress and autophagy were upregulated, whereas proteins involved in FA metabolism were downregulated (Fig. 4). Moreover, excessive TG accumulation was associated with the upregulation of ER stress markers, ROS production, mitochondrial impairment, ATP reductions, and cardiac remodeling and dysfunction in CGI-58^cko^ mice (Figs. 2 and 4–6), whereas inhibition of ER stress by 4-PBA significantly reversed these effects in CGI-58-knockdown NRCMs (Fig. 6) or in CGI-58^cko^ mice (Fig. 7), confirming that ER stress activation was the most important mechanism for CGI-58-deficiency-induced HF. However, the opposite effect of 4-PBA on OCR in NRCMs needs to be verified in future study.

## Conclusion

Here we demonstrated that CGI-58 is a critical regulator for the maintenance of the lipid metabolic balance and cardiac function. CGI-58 knockout in cardiomyocytes induced TG accumulation, activation of ER stress, mitochondrial impairment, and ATP reduction, subsequently leading to cardiac dysfunction. The forced expression of CGI-58 may be a therapeutic strategy for lipid deposit cardiomyovasculopathy. Further studies are required to elucidate the precise mechanism underlying the regulation of CGI-58 expression and the activation of ER stress by CGI-58 in cardiomyocytes.

## Supplementary information

supplemental figure1

supplemental figure2

supplemental figure3

supplemental figure4

supplemental figure legend

## Data Availability

The datasets used during the study are available from the corresponding author upon reasonable request.
