# Correction: Cardiac-specific CGI-58 deficiency activates the ER stress pathway to promote heart failure in mice

**DOI:** 10.1038/s41419-025-07370-0

**Published:** 2025-03-17

**Authors:** Xin Xie, Yi-Fan Tie, Song Lai, Yun-Long Zhang, Hui-Hua Li, Ying Liu

**Affiliations:** 1 Department of Cardiology, Institute of Cardiovascular Diseases, First Affiliated Hospital of Dalian Medical University, Dalian, China; 2 Department of Emergency Medicine, Beijing Key Laboratory of Cardiopulmonary Cerebral Resuscitation, Beijing Chaoyang Hospital, Capital Medical University, Beijing, China

Correction to: *Cell Death and Disease* 10.1038/s41419-021-04282-7, published online 26 October 2021

Since the publication of this paper, the authors have noted that the images of Masson staining for CGI58cKO+saline and CGI58cKO+4-PBA groups in Fig. 7C were shown incorrectly during the manuscript preparation. The correct Fig. 7C is shown below. The authors apologize for the error and state that this correction does not affect the conclusions of the study.

Orginally published figure 7
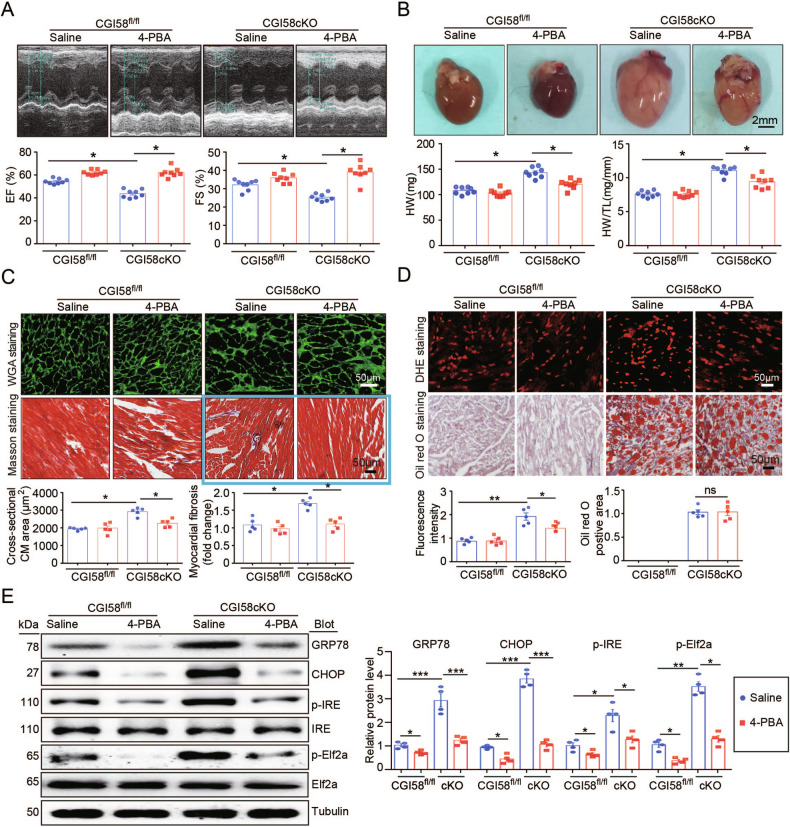


Corrected Figure
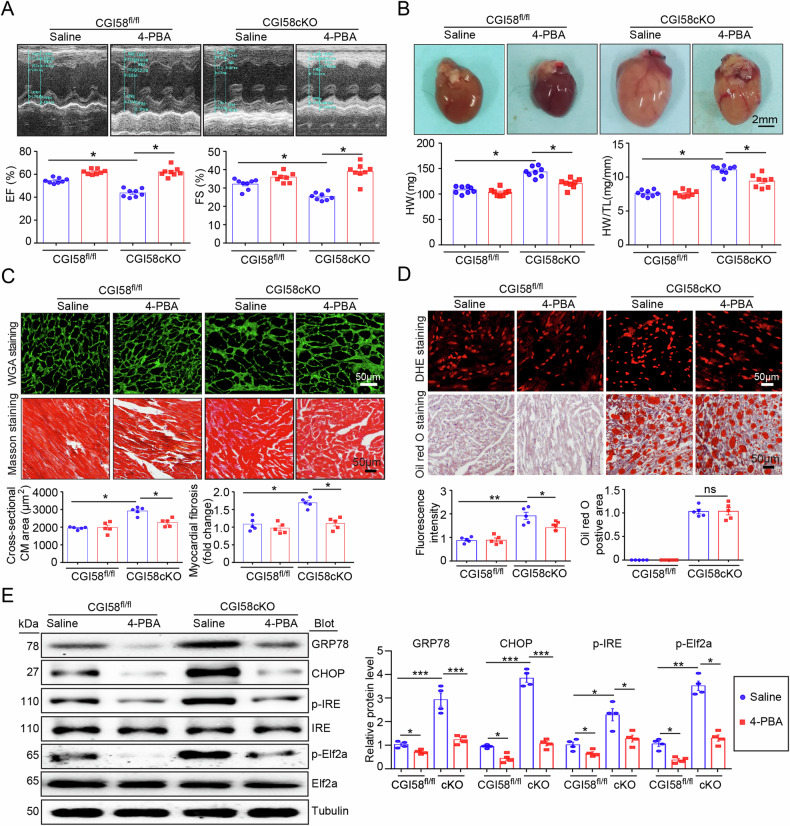

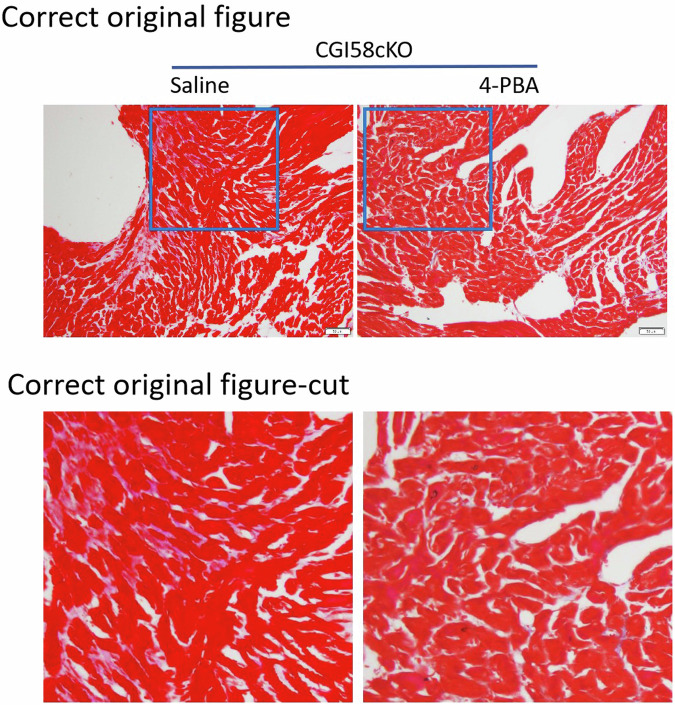


The original article has been corrected.

