# Supplementary figures and images for: Cardiac-specific CGI-58 deficiency activates the ER stress pathway to promote heart failure in mice
Source: Cell Death Dis. 2021 Oct 26;12(11):1003. doi: 10.1038/s41419-021-04282-7 (PMC8548506; doi:10.1038/s41419-021-04282-7)

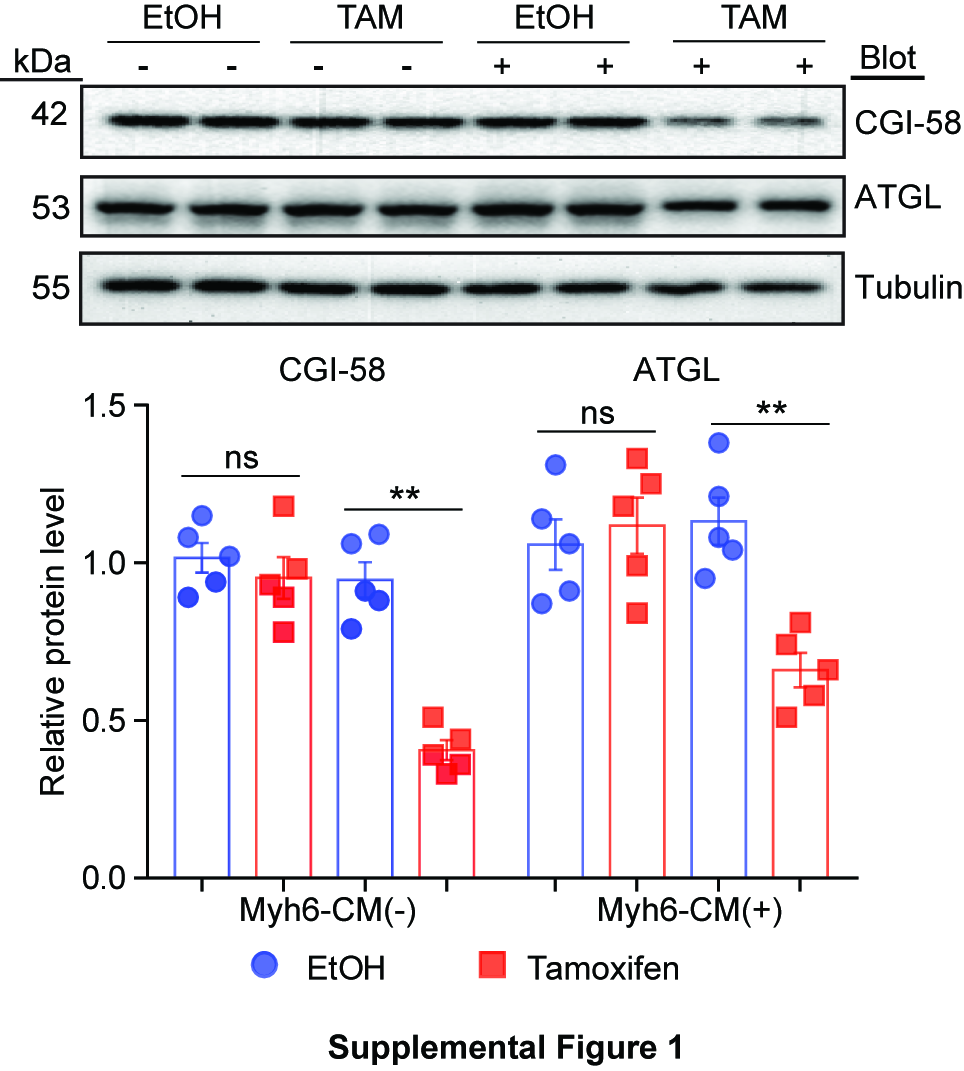

Supplement: Supplementary file 1 — supplemental figure1 [file 41419_2021_4282_MOESM1_ESM.tif]

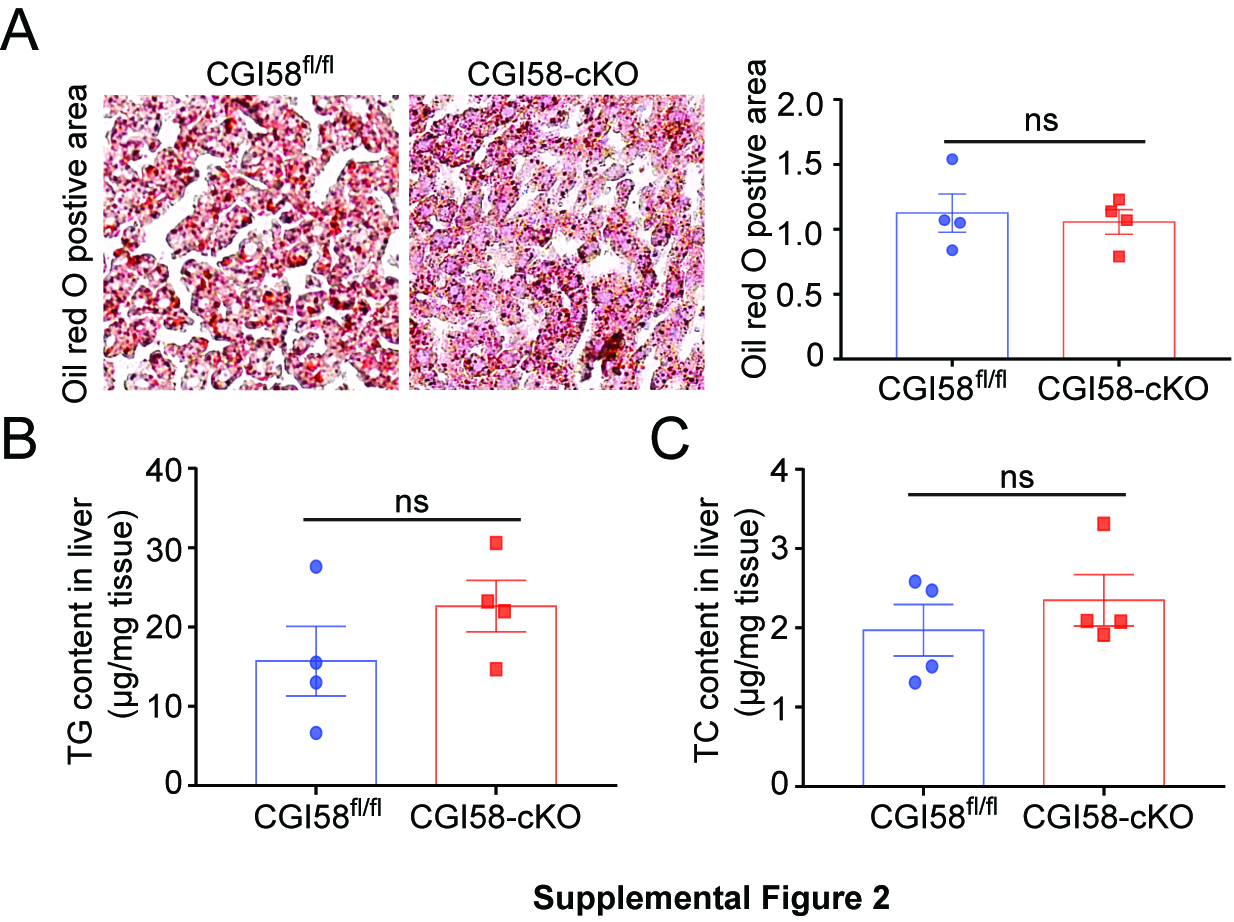

Supplement: Supplementary file 2 — supplemental figure2 [file 41419_2021_4282_MOESM2_ESM.tif]

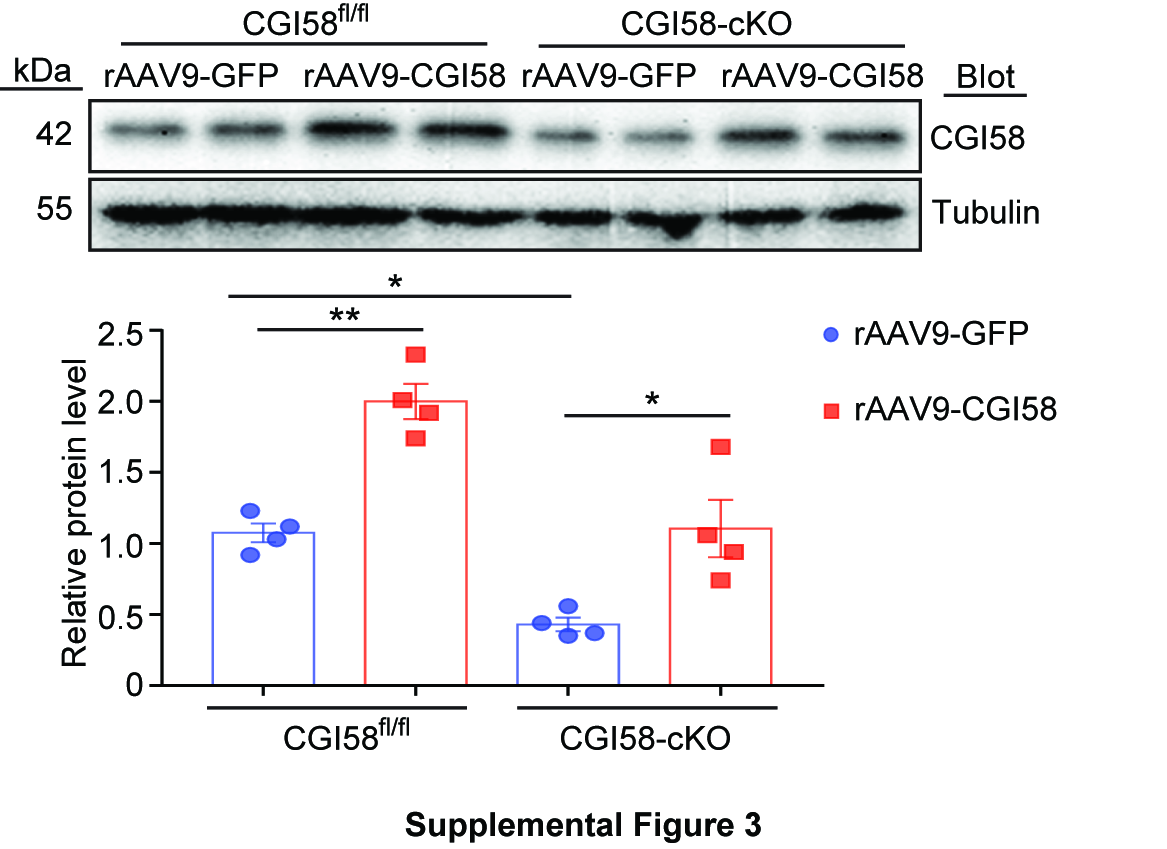

Supplement: Supplementary file 3 — supplemental figure3 [file 41419_2021_4282_MOESM3_ESM.tif]

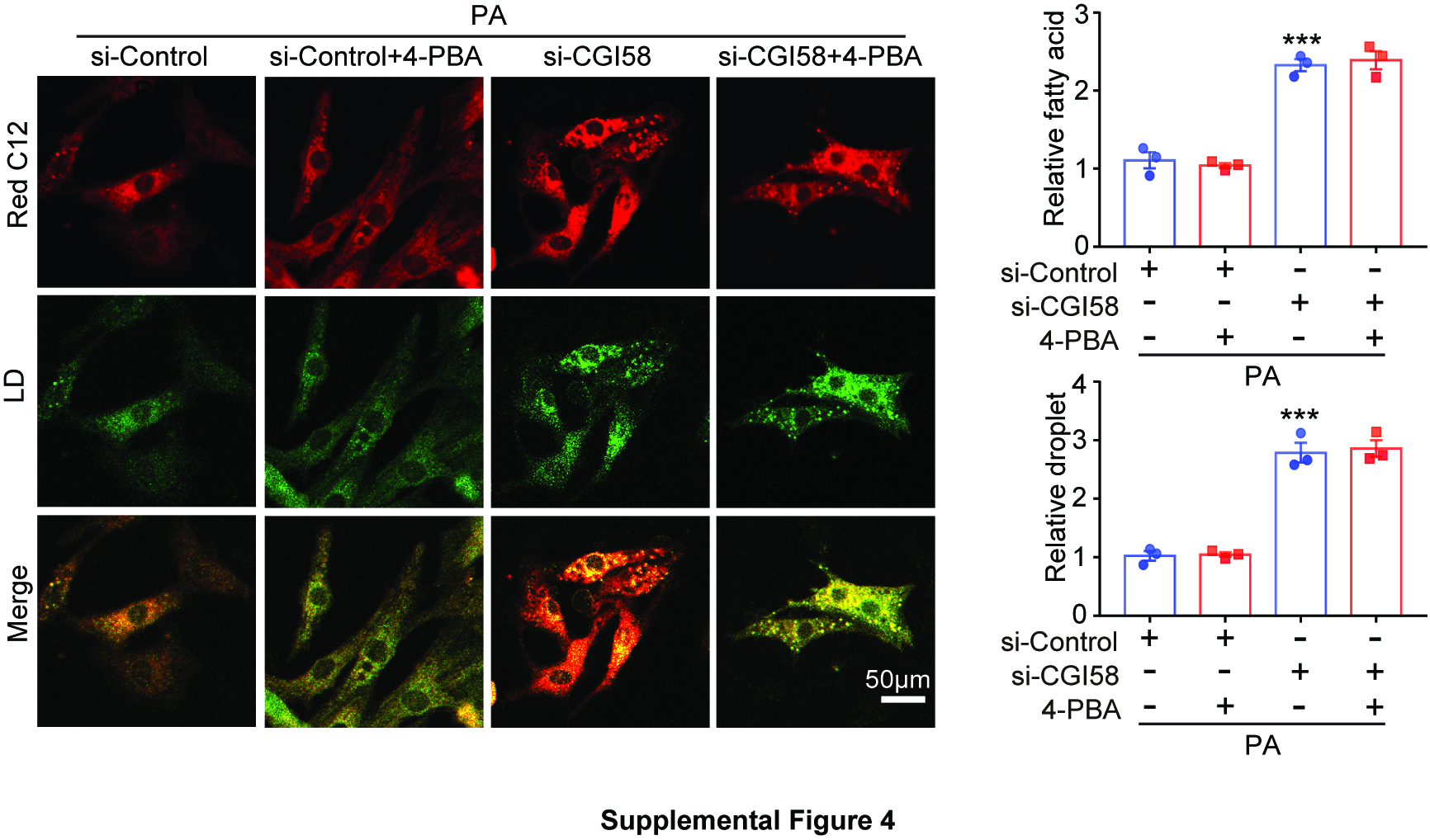

Supplement: Supplementary file 4 — supplemental figure4 [file 41419_2021_4282_MOESM4_ESM.tif]
