# Supplementary material for: Cardiac-specific CGI-58 deficiency activates the ER stress pathway to promote heart failure in mice
Source: Cell Death Dis. 2021 Oct 26;12(11):1003. doi: 10.1038/s41419-021-04282-7 (PMC8548506; doi:10.1038/s41419-021-04282-7)
Supplement: Supplementary file 5 — supplemental figure legend [file 41419_2021_4282_MOESM5_ESM.docx]

**Supplemental figure legends**

**Supplemental Figure 1.** **Tamoxifen injection induces CGI-58 knockdown in heart.** Mice received intraperitoneal injections of tamoxifen (20 mg/kg, dissolved in EtOH) or EtOH once a day for 5 days. After 2 weeks the CGI-58 and ATGL protein expression in the heart were determined by Western blot analysis (upper), and the quantification of relative protein level (lower, n=5). Data are presented as mean ± SEM, and n represents number of animals per group. ***P* < 0.01.

**Supplemental Figure 2.** **Cardiac specific CGI-58 knockout does not affect fatty acid metabolism in liver.** CGI-58^fl/fl^ and CGI-58^cko^ mice were fed with ND for 3 months. (*A*) The lipid accumulation in liver was detected by Oil Red O staining. (*B and C*) Measurement of TG and TC content in the liver (n=4). Data are presented as mean ± SEM, and n represents number of animals per group.

**Supplemental Figure 3. Re-expression of CGI-58 by rAAV9 increases CGI-58 protein level in CGI-58^cko^ mice.** CGI-58^fl/fl^ and CGI-58^cko^ mice receive via tail vein injection 1×10^12^ genome containing particles (gcp) of rAAV9-CGI58 alone or an equivalent amount of empty rAAV9-GFP control for 3 weeks. CGI58 protein level was determined by Western blot analysis (upper) and the quantification of relative protein level (lower, n=4). Data are presented as mean ± SEM, and n represents number of animals per group. **P* < 0.5, ***P* < 0.01.

**Supplemental Figure 4.** **The fatty acid and lipid droplets (LD) accumulation in CGI-58 knockdown NRCMs after PA and 4-PBA treatment.** The NRCMs were treated with BODIPY558/568 C12 (1 μM, Red C12) overnight and BODIPY 493/503 (200 ng/ml) was added to the cells and immediately for imaging. The fatty acid was labeled red and LD was labeled green (left). Scale bar 50 μm. The quantification of fatty acid and LDs (right, n=3 independent experiments). Data are presented as mean ± SEM, and n represents number of animals per group.****P* < 0.001.
